# A comparison between raw and predicted mortality in a paediatric intensive care unit in South Africa

**DOI:** 10.1186/s13104-018-3946-9

**Published:** 2018-11-26

**Authors:** Daynia Elizabeth Ballot, Tanusha Ramdin, Debbie Ann White, Jeffrey Lipman

**Affiliations:** 1 Paediatric/Neonatal Intensive Care Unit, Department of Paediatrics and Child Health, Charlotte Maxeke Johannesburg Academic Hospital, Faculty of Health Sciences, University of the Witwatersrand, Private Bag X 39, 2000 Johannesburg, South Africa; 2 Intensive Care Services, Royal Brisbane and Women’s Hospital and The University of Queensland, Herston, Australia

**Keywords:** Intensive care, Pediatrics, Health care rationing, Mortality audit

## Abstract

**Objective:**

Paediatric intensive care resources are limited in sub-Saharan Africa. The mortality rate in a combined Paediatric/Neonatal Intensive Care Unit in Johannesburg, South Africa was almost double that in a dedicated paediatric intensive care unit in the same country. This study aimed to compare the raw mortality rate with that predicted with the Paediatric Index of Mortality (version 3), by doing a retrospective analysis of an existing database.

**Results:**

A total of 530 patients admitted to the intensive care unit between 1 January 2015 and 31 December 2017 were included. The raw mortality rate was 27.1% and the predicted mortality rate was 27.0% (p = 0.971). Cardiac arrest during ICU admission (p < 0.001), non-reactive pupils (0.035), inotropic support (p < 0.001) and renal disease (p = 0.002) were all associated with an increased risk of mortality. These findings indicate that the high mortality rate is due to the severity of illness in the patients that are admitted. It also indicates that the quality of care delivered is acceptable.

## Introduction

Paediatric intensive care units (PICUs) are a scarce resource in low and middle income countries (LMICS), particularly sub-Saharan Africa. The majority of deaths in children below 5 years of age occur in LMICS, yet health care resources and per capita health care expenditure are greater in high income countries (HIC) [1–3]. South Africa is a middle income country with limited resources and a shortage of ICU beds. A descriptive study in South Africa in 2007 showed that only 23% of public sector hospitals had ICU or high care beds, only 19.6% of the available ICU beds were dedicated to paediatric or neonatal patients [4]. In resource constrained settings, admission to PICU is frequently limited to those patients with the best anticipated outcome. The ethical principle of social or distributive justice applies [5].

The mortality rate in paediatric ICU can provide an indication of the quality of care and guide allocation of scarce resources. A review of the current study unit, which is a combined Paediatric/Neonatal Intensive Care Unit (PNICU) in Johannesburg, South Africa, showed that 59% of admissions were neonates, 27% had primary surgical conditions and 14% were for primary medical conditions [6]. Neonatal care in the same PNICU has been previously described [7–10]. The mortality rate of paediatric patients (older than 28 days) in the current study PNICU was higher than the mortality rate reported from the dedicated PICU at Red Cross War Memorial Children’s Hospital (RCWMCH) [10, 11]. Different units should be compared with mortality rates adjusted for severity of illness. The Paediatric Index of Mortality score (PIM) is an example of a severity of illness score which has been shown to be predictive of mortality in paediatric patients in HIC [12]. The PIM has previously been validated in validated in South Africa [13].

The current study aimed to determine compare the raw and predicted mortality rates for paediatric admissions to a combined PNICU in Johannesburg, South Africa.

## Main text

### Subjects and methods

This was a retrospective review of all paediatric patients (1 month to 14 years of age) admitted to a combined PNICU in Johannesburg, South Africa, between 1 January 2015 and 31 December 2017. Only the first admission was included in the analysis, recurrent admissions were excluded. Patients without a recorded outcome (died or survived) were also excluded.

#### Paediatric Neonatal Intensive Care Unit

The PNICU admitted children of all ages (from birth to 14 years) and for both surgical and medical indications [10], although only paediatric patients (above 1 month of age) were included in this review. Patient characteristics and outcome have been reported in detail previously [10]. There were 14 ventilator beds during the study period and the unit essentially functioned as a ventilator unit, not as a high care or observation unit. The hospital offered specialized paediatric care and specialized referrals were received from the whole region, including neighboring southern African countries. The unit admission guidelines were based on the Royal Paediatrics and Child Health guidelines [14]. Children who were brain dead and those considered to have a very poor chance of intact survival were not routinely admitted. Neonatologists, a pulmonologist and a gastroenterologist cared for the patients, there was no dedicated paediatric intensivist. Nurse to patient ratio was one nurse to two infants below 3 months old and one nurse to one older patient. Monthly mortality audits were conducted during the study period.

#### Data collection

The study was a secondary analysis of an existing database. Demographic and clinical information was collected on discharge from the unit for each patient and entered into a computer database. Data was managed using Research Electronic Data Capture, (REDCAP), hosted by the University of the Witwatersrand [15]. Data required for the calculation of the PIM 3 was collected on admission of each patient [12].

#### Statistical analysis

IBM SPSS version 25 was used for analysis. Continuous variables were described using mean and standard deviation or median and interquartile range categorical variables were described using frequencies and percentages. Patients who survived to discharge from PNICU were compared to non-survivors. Univariate analysis was done using Chi square analysis to compare categorical variables while unpaired t tests or non- parametric tests were used for continuous variables as appropriate. A p value of < 0.05 was considered to be statistically significant. Only valid cases were included in the analysis—i.e. missing variables were omitted for each variable. Those variables found to be statistically significant on univariate analysis were entered into a multivariate logistic regression with death in ICU as the outcome variable, in order to determine adjusted odds ratios for significant predictors of mortality.

Bacterial, viral and fungal infections were defined as isolation of a pathogen in a sterile body fluid– either by culture or on Polymerase chain reaction (PCR). As part of the routine prevention of mother to child transmission of HIV infection, children born to HIV infected mothers were routinely screened with a PCR at birth and 10 weeks of age; infected children were commenced immediately on antiretroviral therapy [16]. Only children who were HIV exposed and presented to the PNICU with signs and symptoms suggestive of HIV infection would be tested with a PCR. There was no routine screening for HIV on admission to PNICU. Cardiac arrest prior to PNICU admission was classified as “post cardiac arrest” as opposed to patients who had a cardiac arrest during the course of the PNICU admission.

The predicted mortality rate was calculated using the PIM 3 calculator (https://www.anzics.com.au/wp-content/uploads/2018/08/PIM3-Calculator.xlsx) Data collected on admission for the calculation of the PIM 3 score included: systolic blood pressure (mmHg) (uncorrected for age) pupillary reaction to bright light; FiO_2_ and PaO_2_ (mmHg); base excess on arterial or capillary blood sample (mmol/L), elective admission; admission for recovery post-procedure; low risk diagnosis, high risk diagnosis and very high risk diagnosis [12]. Only patients with complete PIM data were included in this calculation. A single sample t test was used to compare this mortality rate with the actual mortality rate.

### Results

There were 602 paediatric admissions to the PNICU during the study period, 68 were recurrent admissions, so were excluded and four patients did not have an outcome recorded. The final sample therefore consisted of 530 paediatric patients admitted to the PNICU. The median age on admission was 256 days (IQR 1522). The median duration of admission to the PNICU was 3 days (IQR 5). There were 147 deaths in the PNICU (27.7%). There were 49/530 (9.2%) patients admitted after cardiac arrest, of whom 31/49 (63.2%) had lower respiratory tract infections (LRTI). Cardiac arrest during PNICU admission occurred in 88/530 (16.6%); 38/88 (43.2%) had LRTI. Most patients (81/88; 92%) who had a cardiac arrest during admission died, compared to 31/49 (63.2%) who were admitted post cardiac arrest (p < 0.001). The admission diagnosis was classified as high risk in 51/534 (9.6%) and very high risk in 65/524 (12.2%). Univariate analysis on the clinical and demographic characteristics associated with mortality are shown in Tables 1 and 2.

**Table 1:** Clinical and demographic characteristics associated with mortality in paediatric intensive care patients

Variable	Total n/N (%)	Survived n/N (%)	Died n/N (%)	p value
Male sex	278/521 (53.4)	203/374 (54.3)	75/147 (51.0)	0.502
Elective admission	100/530 (18.90	92/383 (24.0)	8/147 (5.4)	< 0.001
Recovery	107/530 (20.2)	100/383 (26.1)	7/147 (4.8)	< 0.001
HIV exposed	108/463 (23.3)	65/337 (19.3)	43/126 (34.1)	0.001
HIV PCR positive^a^	29/103 (28.2)	17/64 (26.6)	12/39 (30.8)	0.645
Post cardiac arrest	49/527 (9.3)	18/380 (4.7)	31/147 (21.1)	< 0.001
Cardiac arrest in ICU	88/522 (16.9)	7/381 (1.8)	81/141 (57.4)	< 0.001
Septic shock	47/530 (8.9)	13/383 (3.4)	34/147 (23.1)	< 0.001
Chromosomal abnormality	13/530 (2.5)	7/383 (1.8)	6/147 (4.1)	0.133
Pupils fixed and dilated	24/427 (5.6)	6/310 (1.9)	18/117 (15.4)	< 0.001
Surgical patient	287/514 (55.8)	229/370 (61.9)	58/144 (40.3)	< 0.001
Post-operative	253/522 (48.5)	214/379 (56.5)	39/143 (27.3)	< 0.001
Trauma patient	88/530 (16.6)	60/383 (15.7)	28/147 (19.0)	0.349
Non accidental injury	21/530 (4.0)	15/383 (3.9)	6/147 (4.1)	0.930
Patient intubated and ventilated	487/528 (92.2)	343/382 (89.8)	144/146 (98.6)	0.001
Inotropic support	118/527 (22.4)	36/382 (9.4)	82/145 (56.6)	< 0.001
Upper respiratory tract	45/530 (8.5)	38/383 (9.9)	7/147 (4.8)	0.056
Lower respiratory tract	186/530 (35.1)	129/383 (33.7)	7/147 (38.8)	0.271
Cardiovascular	99/530 (18.7)	52/383 (13.6)	47/147 (32.0)	< 0.001
Neurology	137/530 (25.8)	94/383 (24.5)	43/147 (29.3)	0.268
Gastrointestinal	126/530 (23.8)	89/383 (23.2)	37/147 (25.2)	0.640
Renal	80/530 (15.1)	40/383 (10.4)	40/147 (27.2)	< 0.001
Haematology oncology	99/530 (18.7)	69/383 (18.0)	30/147 (20.4)	0.527
Metabolic problems	129/530 (24.3)	60/383 (15.7)	69/147 (46.9)	< 0.001
Poisoning	17/522 (3.3)	10/380 (2.6)	7/142 (4.9)	0.188
Bacterial sepsis^b^	89/523 (17.0)	50/379 (13.2)	39/144 (27.1)	< 0.001
Viral infection (excluding HIV)^b^	36/513 (7.0)	24/372 (6.5)	12/141 (8.5)	0.415
Fungal infection^b^	17/507 (3.4)	8/369 (2.4)	8/138 (5.8)	0.062

^a^Only HIV exposed patients with signs suggestive of AIDS were tested with HIV PCR

^b^Proven sepsis (organism isolated on culture or PCR)

**Table 2:** Admission characteristics of survivors and non survivors in paediatric intensive care patients

Variable	Total group	Survived	Died	p value
Systolic blood pressure (mmHg)	97.87 (25.07)	100.77 (23.56)	90.71 (27.37)	< 0.001
FiO_2_	0.71 (0.26)	0.67 (0.26)	0.80 (0.26)	< 0.001
PaO_2_ (mmHg)	111.75 (60.43)	115.55 (60.68)	102.19 (58.96)	0.051
Base Excess (mmol/L)	− 5.48 (14.86)	− 4.16 (9.9)	− 8.94 (22.98)	0.006

All values expressed as mean and standard deviation

There were 450 patients with complete PIM 3 data. The raw mortality rate in this sub-group was 27.1% and the predicted mortality rate using the PIM 3 calculation was 27.0% (p = 0.971).

The median admission age of patients was not statistically different between survivors and non-survivors [248 days (IQR 1411) vs 257 days (IQR 1569) p = 0.23]. Similarly, there was no significant difference in the median duration of the PNICU admission between survivors and non-survivors [3.0 days (IQR 3.0) vs 3 days (IQR 6.0) p = 0.702]. The adjusted odds ratios for factors associated with mortality are shown in Table 3. Cardiac arrest during PNICU admission, fixed dilated pupils, inotropic support and renal disease were all associated with an increased risk of mortality, while post-operative patients and systolic blood pressure on admission had a reduced mortality.

**Table 3:** Adjusted odds ratios for clinical characteristics associated with mortality in paediatric intensive care patients

Variable	Odds ratio	95% Confidence intervals	p value
Cardiac arrest during admission	22.11	8.27–59.11	< 0.001
Pupils fixed and dilated	5.61	1.13–27.80	0.035
Post-operative patient	0.26	0.12–0.56	0.001
Inotropic support	7.56	3.45–16.12	< 0.001
Renal system	4.39	1.76–10.99	0.002
Systolic blood pressure	0.98	0.97–0.99	0.005

### Discussion

This review of over 500 paediatric ICU patients in a combined PNICU in a tertiary referral hospital in South Africa, found a mortality rate of 27.7%. The mortality rate was considerably higher than that reported from a dedicated PICU in the same country [11]. However, the predicted mortality rate using the PIM3 score in this PNICU was the same as the raw mortality rate, reflecting a high burden of disease. Almost one quarter of the paediatric admissions had a high or very high risk diagnosis indicating that seriously ill children with a poor chance of survival were often admitted to the PNICU during the study period. Reasons for this were not evaluated, but may reflect the lack of a strict admission protocol, absence of a paediatric intensivist and relative inexperience of many of the attending staff. Previous research has demonstrated that the outcome of critically ill children is best if they are treated in dedicated PICU with paediatric intensivists in attendance [17, 18]. This is, however, unlikely to be achieved in most LMICS due to resource limitations.

The lack of difference in the raw and predicted mortality rates gives a crude indication that the standard of care in the unit was acceptable. Inferior patient care would be reflected in a predicted mortality rate that was much lower than the actual rate.

The majority of admissions in the current study were surgical. Post-operative cases had a significantly lower risk of mortality, suggesting that the use of PICU for post-operative care of elective cases is beneficial. This is in agreement with Argent et al. who argue that delay of elective surgical cases can result in complications with a worse outcome [11].

Cardiac arrest during the PNICU admission, non-reactive pupils on admission, the need for inotropic support and renal system disease were associated with an increased chance of mortality in the current study. Cardiac arrest in children has generally been considered to have a poor outcome, but about one quarter of children resuscitated for in-hospital cardiac arrest will survive to hospital discharge [19]. Admission to PICU post resuscitation for cardiac arrest should therefore take into consideration the need for inotropic support and whether the patient’s pupils are reactive. Although renal system disease was associated with an increased risk of mortality in the present study, renal replacement therapy-including kidney transplant was available in the study hospital. In this context, it would therefore not be justified to exclude children in kidney failure from admission to ICU. Other prognostic factors should be determined in these children.

Hematology oncology patients did not have a higher chance of death in the PNICU. Admissions for children with cancer were most commonly post-operative. Other children with cancer were only admitted if they were considered to have a reversible condition and to be responding well to the cancer therapy [20].

### Conclusion

The mortality rate in the current study was high, but the raw and predicted mortality rate was the same, suggesting that although severely ill children were admitted, the level of care was acceptable. PNICU. Admission criteria should be developed using locally obtained data to maximize the benefit of limited PNICU resources.

### Implications of the study

The results of the current study emphasize the importance of correcting mortality rates for severity of illness, particularly when comparing different units. The challenge of restricted PNICU facilities in LMICS is compounded by a high burden of disease.

## Study limitations

This was a retrospective secondary analysis of an existing database so not all data was complete. Information, such as treatment protocols, staffing ratios, the availability of equipment and the impact of these factors on survival rates could not be analyzed. Decisions to admit patients was at the discretion of the attending doctor, so admission criteria were not standardized. There was no information on the number of children who were refused admission to the PNICU and their outcomes.

## Abbreviations

HIC: high income country
HIV: human immunodeficiency virus
ICU: intensive care unit
LMICS: low and middle income countries
NCPAP: nasal continuous airways pressure
PICU: paediatric intensive care unit
PIM: Paediatric Index of Mortality
PNICU: Paediatric/Neonatal Intensive Care Unit
RCWMCH: Red Cross War Memorial Children’s Hospital
REDCAP: Research Electronic Data Capture
